# Live culture-based qPCR screening of Taq DNA polymerase variants for resistance to PCR inhibitors

**DOI:** 10.3389/fbioe.2025.1624735

**Published:** 2025-08-29

**Authors:** Milko B. Kermekchiev, Zhian Zhang, Wayne M. Barnes

**Affiliations:** ^1^ DNA Polymerase Technology, Inc., St. Louis, MO, United States; ^2^ Biochemistry and Molecular Biophysics, Washington University School of Medicine, St. Louis, MO, United States

**Keywords:** life culture PCR (LC-PCR), enzyme screening, mutagenesis, PCR inhibitor, Taq polymerase

## Abstract

We present a live culture PCR (LC-PCR) workflow that enables direct screening of randomly mutagenized Thermus aquaticus (Taq) and Klentaq1 DNA polymerase libraries without enzyme purification. Intact bacterial cells expressing individual variants serve as both enzyme source and DNA template in real-time PCR, allowing rapid selection for inhibitor resistance in a 96-well format. Screening ~14,000 clones in the presence of potent inhibitors (chocolate, black pepper) yielded two novel variants—Taq C-66 (E818V) and Klentaq1 H101 (K738R)—with superior resistance to diverse PCR inhibitors, including blood, humic acid, and plant extracts, compared to wild-type and previously known resistant mutants. Resistance persisted after purification, indicating intrinsic enzymatic tolerance. Structural mapping suggests these substitutions may enhance nucleotide binding or stabilize the polymerase—DNA complex, reducing susceptibility to inhibitor interference. LC-PCR reduces screening time, cost, and contamination risk, and is readily adaptable for evolving polymerases with enhanced speed, fidelity, or substrate versatility.

## Introduction

For more than three decades, PCR technology has provided essential tools for genome and transcriptome studies, as well as important applications in clinical, environmental, food safety, and forensic analyses. The essential component in PCR is a thermostable DNA polymerase, such as Taq pol, capable of multiple rounds of DNA amplification *in vitro*. The broad variety of PCR applications and related challenges impose ongoing demands on this enzyme, such as resistance to inhibitors, sensitivity, specificity, fidelity, and elongation speed. A major concern regarding Taq is its low tolerance to a broad spectrum of PCR inhibitors found in clinical, soil, food, plant, forensic, and other matrices, which can cause false-negative results.

Various DNA extraction protocols and kits are employed to purify DNA prior to PCR, but these methods are costly, time-consuming, and may not fully remove inhibitors. Additionally, they may introduce contamination and lead to DNA or RNA losses. There are continuous efforts to improve Taq performance, often achieved by directed evolution. This involves selecting beneficially modified forms of the enzyme from a library containing numerous clones of randomly mutagenized Taq genes. Diversification of the whole gene or specific catalytic domains can be achieved through methods such as error-prone PCR, DNA shuffling, or *in vivo* methods (Cadwell and Joyce, 1992; Hanson-Manful et al., 2013; Stemmer, 1994; Muteeb et al., 2010; Packer and Liu, 2015).

A reliable and efficient functional screening/selection assay is critical for the successful selection of desirable Taq variants (reviewed in (Chen and Romesberg, 2014)). Some previously described screening procedures involve radiolabeled nucleotide incorporation in in situ colony assays (Sag et al., 1991; Kermekchiev et al., 2003), or DNA polymerase I *in vivo* complementation assays (Suzuki et al., 1996), and phage display assays (Xia et al., 2002). However, these approaches are either time-consuming, complex, or not fully reliable for selecting Taq mutants resistant to inhibitors. More recently, a library screening approach was employed, where crude cell lysates from individual library clones are used in fluorescence primer extension or amplification assay in the presence of SYBR green (Gloeckner et al., 2010; Drum et al., 2014). The procedure requires at least two hours of pre-PCR steps, including a two-step heating for lysing the cells and inactivation of non-Taq bacterial proteins and double centrifugation, which adds some risk of cross-contamination. In a different concept for selection of Taq mutants, called compartmentalized self-replication (CSR), individual Taq library clones are isolated from each other in water/oil emulsion compartments, thus they replicate only their own encoding gene. This allows a positive selection of mutant clones with the desired phenotype (Ghadessy et al., 2001; Baar et al., 2011). The emulsion quality and the size of the formed particles is of importance, and its control requires special equipment, such as a particle analyzer or a microscope.

In this study, we describe a simplified library screening procedure using intact bacterial cells still in culture, expressing individual enzyme clones of mutagenized Taq or Klentaq1 libraries. These cells serve directly as the PCR enzyme in real-time PCR, performed in a microtiter plate format. This procedure eliminates pre-PCR enzyme purification steps, making it time-saving and cost-efficient. We demonstrate its application with the selection and characterization of Taq pol and Klentaq1 mutant enzymes that exhibit high resistance to PCR inhibitors found in blood, chocolate, black pepper, plant tissues, and humic acid.

## Materials and methods

### Bacterial strains, expression vectors, and PCR primers

Codon numbers correspond to wild-type (full-length Taq pol) *Thermus aquaticus* DNA polymerase (832 amino acids) (Lawyer et al., 1989). Klentaq1 is an N-terminal deletion of 278 amino acids (Bar and nes, 1992). pUC18 and pWB254 were used as expression vectors for Taq and Klentaq1 libraries, respectively. Bacterial strains *E. coli* and X7029 were used to express and purify Taq and Klentaq1 enzyme variants. PCR primers for 16S rRNA and other targets are listed in Table 1.

**TABLE 1:** Primers and templates.

Experiment	Template	Forward primer	Reverse primer
LC-PCR Screen	16S rRNA gene	C-For: 5′-TACAGACGTTTGAGCTTCGCAATTACCGGTT-3′ Q-For: 5′- CCACCAGACGATAGTTATCACGCA-3′ U-For: 5′-AGGAGGTGATCCAACCGCA-3′	C-Rev: 5′-AAAAAGCTGCAAATTGCGGTAGGTATTATT-3′ Q-Rev: 5′-CTGATCGAATGGCTGCCAGGCTCC-3′ U-Rev: AACTGGAGGAAGGTGGGGAT-3′
LC-PCR Amplification	Human beta-actin	bAC-For: 5′-CAG​CGG​AAC​CGC​TCA​TTG​CCA​ATG​G-3′	bAC-Rev: 5′-TCA​CCC​ACA​CTG​TGC​CCA​TCT​ACG​A-3′
Inhibition-resistance Test	*Salmonella*	Hil-A3-For: 5′-AGT​TGG​AGG​AGG​CCT​TAC​AAA​CGA-3′	Hil-A3-Rev: 5′-TAT​CCT​GCA​GGT​GTT​GTG​AGC​GTA-3′
Blood-resistance Test	Human HIV receptor CCR5	KOZ (common forward primer): 5′-TGG​AAC​AAG​ATG​GAT​TAT​CAA​GTG​TCA​AGT​CCA-3′	CCR5-0.63 kb: 5′-GCAGCGGCAGGACCAGCCCCAAGATGACTATCT-3′ CCR5-1.1 kb: 5′-AGGCTGTGTATGAAAACTAAGCCATGTGCACAA-3′
Inhibition-resistance Test	Soybean Cyst Nematode	SCN2-For: 5′-CAT​TCT​CCG​CGA​CAC​CGT​AAT-3′	SCN2-Rev: 5′-CAT​CTT​CCC​ATC​CAA​CAC​CG-3′

### PCR performed with growing cells (live culture PCR)

One to five mL of host bacterial cells (*E. coli* or X7029) expressing Taq, OmniTaq, or OmniKlentaq were induced with 1 mM IPTG for 12–16 h at 37 °C in an orbital shaker and used directly in PCR. Two to five µL of cell culture were added to a master mix containing PCR buffer (50 mM Tris-HCl, pH 9.2, 2.5–3.5 mM magnesium chloride, 16 mM ammonium sulfate, and 0.025% Brij-58), dNTPs at 250 µM each, and primers, followed by immediate PCR cycling. For amplification of endogenous targets, universal (“U”) 16S rRNA gene (rDNA) primers were used, with bacterial DNA from the cells serving as the template. For exogenous targets, primers for the human beta-actin gene and 10 ng of human DNA were added. PCR was carried out for 35–45 cycles, with cycling conditions optimized for standard PCR using purified enzyme and template, including an extended initial denaturation step of 8–10 min at 94 °C. Induced bacterial cultures were stored at 4 °C and could be reused for PCR for at least several days, as they retained polymerase activity.

### Library preparation and LC-PCR screening

Randomly mutagenized libraries of Taq and Klentaq1 were prepared by error-prone Mg/Mn PCR, as previously described (Cadwell and Joyce, 1992; Kermekchiev et al., 2003). The amplified products were cloned into pUC18 or pWB254 expression vectors. Single colonies were picked from bacterial host cells (*E. coli* and X7029) transformed with the recombinant plasmids and placed in individual wells of U-bottom 96-well plates containing 100 µL of Amp+ media and 1 mM IPTG.

In later screenings, single-colony picking was eliminated, and cultures were diluted to approximately 1 cell per 10–20 µL. The plates were incubated for 12–16 h at 37 °C in an orbital shaker at 100–150 rpm for bacterial growth and enzyme induction. Five µL of culture from each well were transferred to a replica (PCR) 96-well plate, with each well containing 30 µL of PCR master mix. The master mix included buffer (as described above), dNTPs at 250 µM each, rDNA U-primers, 0.5X SYBR Green, 0.5X PEC-1 enhancer (DNA Polymerase Technology), and the challenging PCR inhibitor (2–3 µL of 10% chocolate or 10% black pepper extract per 35 µL reaction). Replica plates were immediately subjected to real-time PCR using the same cycling conditions as for single-reaction LC-PCR of rDNA: 94 °C for 10 min, followed by 40–45 cycles of 94 °C for 30 s, 54 °C for 40 s, and 70 °C for 2 min.

Corner wells of each PCR plate served as controls and contained cells expressing OmniTaq or OmniKlentaq, our previously reported inhibition-resistant Taq mutants (Kermekchiev et al., 2009; Zhang et al., 2010). No enzyme or DNA were added, as these were provided by the library cells. The primary plates, used to grow and induce library clones, were stored at 4 °C for up to 5–6 days. During this period, they could be reused multiple times for re-screening without significant loss of polymerase activity. Stocks of the entire plate or selected clones were made by adding glycerol to 12% v/v and freezing at −80 °C. After single-colony purification, open reading frames (ORFs) were amplified and both DNA strands were cycle-sequenced with overlapping reads using dye-ddNTPs (Genewiz).

### Purification and functional testing of selected mutants

For initial testing of the selected mutants, small-scale purification of the enzymes was performed as previously described (Kermekchiev et al., 2003; Kermekchiev et al., 2009), with the procedure scaled down to 50 mL of starting bacterial cultures. After confirming the selected phenotypes in PCR inhibition assays, the same purification protocol was applied to large-scale enzyme preparations, starting with 7–14 L of induced cultures.

Inhibition resistance tests were conducted with increasing amounts of various inhibitors, including chocolate, black pepper, blood, corn leaf extract, humic acid, and others. In parallel reactions, wild-type (wt) Taq DNA polymerase (New England Biolabs) was used as a reference enzyme. Dark chocolate (70% cocoa) and black pepper powder were purchased from grocery stores, and 10% water solutions of each were prepared by heating the mixture at 75 °C for 20 min and suspending the material with a bead beater. Human whole blood (EDTA-treated) was purchased from Valley Biomedical. Maize leaf extract was prepared by homogenizing corn leaf pieces in water at a 1:5 ratio using a bead beater, followed by centrifugation. Humic acid was obtained from Dr. S. Sinha (InnoGenomics) as part of a collaborative project.

**FIGURE 1:**
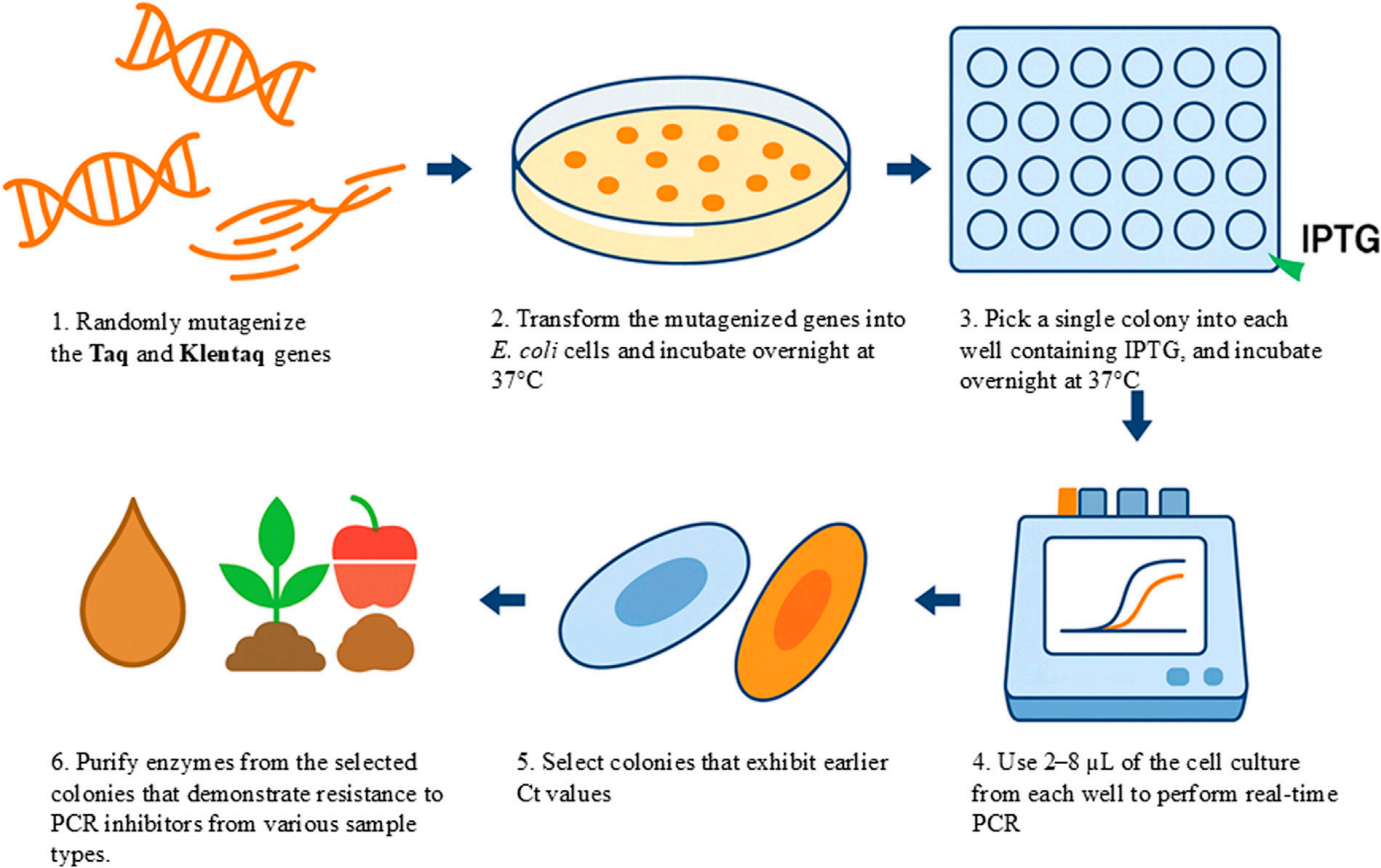
A graphical representation of the “live culture” PCR (LC-PCR) screening workflow. Mutant libraries of DNA polymerase are generated and transformed into host cells, which are plated on agar to form colonies. Colonies are transferred into microtiter plates and induced with IPTG for protein expression. Life-cells are then subjected to real-time PCR assays in the presence of various PCR inhibitors. High-performing mutants exhibiting strong amplification under inhibitory conditions are selected and further validated using crude samples such as soil, plant matter, or food products without prior DNA purification.

A schematic representation of the screening workflow is shown in Figure 1.

## Results

### Direct DNA amplification with bacterial cell cultures expressing wild-type Taq and Taq variants

To explore the possibility of performing PCR in an extremely simplified system where the enzyme and template are provided directly by bacterial cells expressing a thermostable DNA polymerase, we performed a series of experiments. In these experiments, mini cultures of cells expressing previously identified inhibition-resistant Taq mutants, (OmniTaq (OT) and OmniKlentaq (OKT)) were IPTG-induced and added last to the PCR mix. OT and OKT were previously characterized as inhibition-resistant Taq mutants (Kermekchiev et al., 2009; Zhang et al., 2010).

**FIGURE 2:**
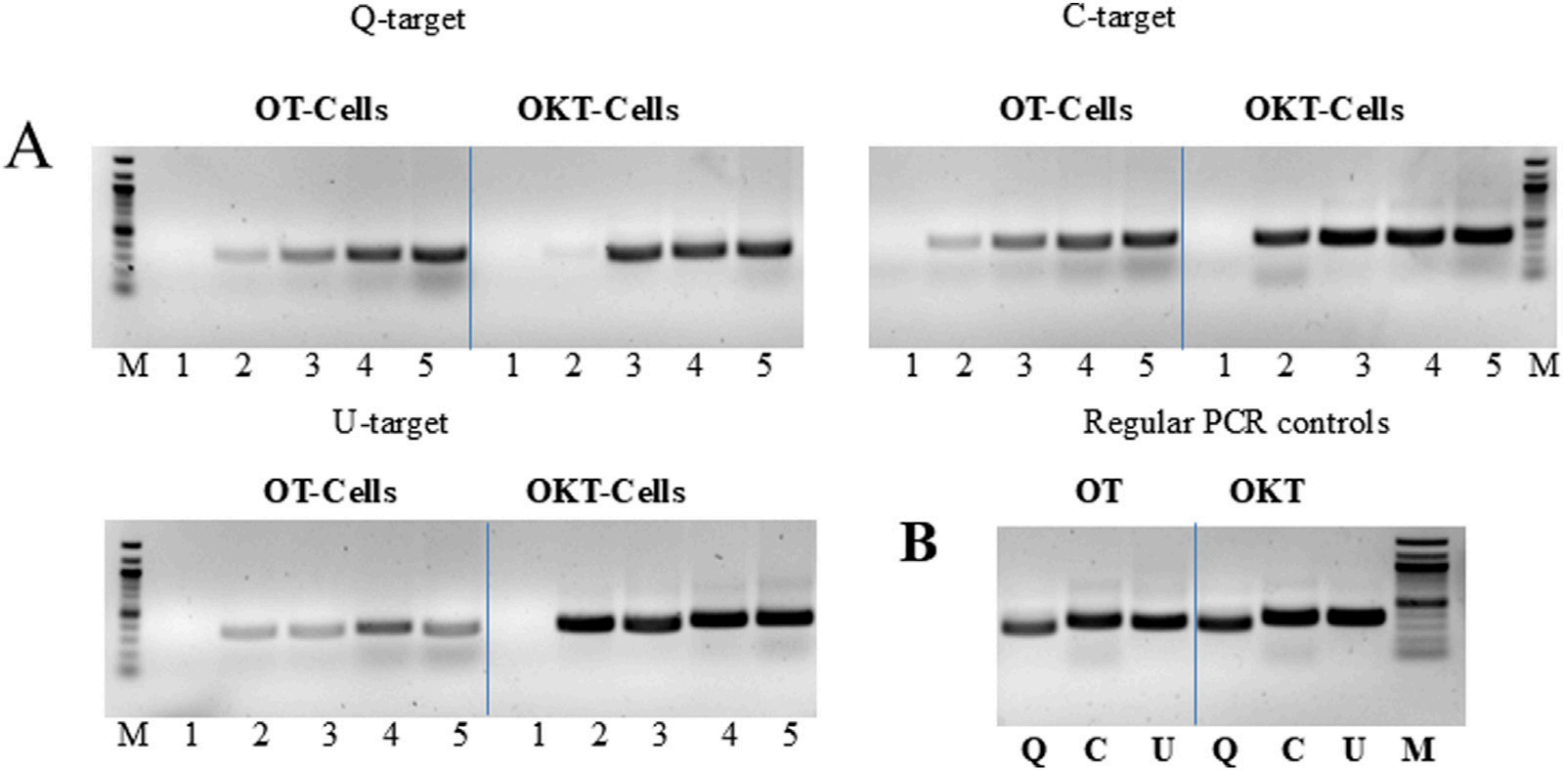
“Live Culture PCR” performed with cells expressing two mutant versions of Taq. **(A)** Three endogenous 16S rRNA gene (rDNA) targets (Q, C, U) were amplified by PCR, using bacterial cells expressing OmniTaq (OT) or Omni Klentaq (OKT) DNA polymerase as the catalyst. Reactions contained 0, 1, 2, 4, or 8 µL of culture per 50 µL reaction (lanes 1–5). **(B)** Positive controls: conventional PCR was performed with 1 ng of total bacterial DNA and 0.1 µL of purified OT or OKT enzyme, using the same primers for targets Q, C and U (lanes Q, C, U). Reactions contained no PCR enhancer or other additives. Amplified products were analyzed by 1.5% agarose gel electrophoresis, stained with ethidium bromide. Lanes M, 100 bp DNA ladder.

We attempted to amplify endogenous bacterial targets, such as rDNA (targets U, C and Q), to determine whether both the enzyme and template could be provided by the host cells. The PCR mix only contained the buffer, primers, and deoxyribonucleoside triphosphates (dNTPs). The results showed that cells expressing the OT and OKT polymerases could indeed provide sufficient enzyme and template for efficient and specific PCR. Amplification was observed with as little as 1–2 µL of cell culture, and the amplification level was positively correlated with the culture volume in the range of 1–8 µL (Figures 2A,B). Cell expressing OmniKlentaq provided higher amplification level as compared to OmniTaq, consistent with earlier studies showing that the Klentaq1 is more robust relative to the full-length Taq, which is generally true for the mutants derived from both (Kermekchiev et al., 2009; Zhang et al., 2010). This was surprising, considering possible complications expected from enzyme impurity and degradation.

**FIGURE 3:**
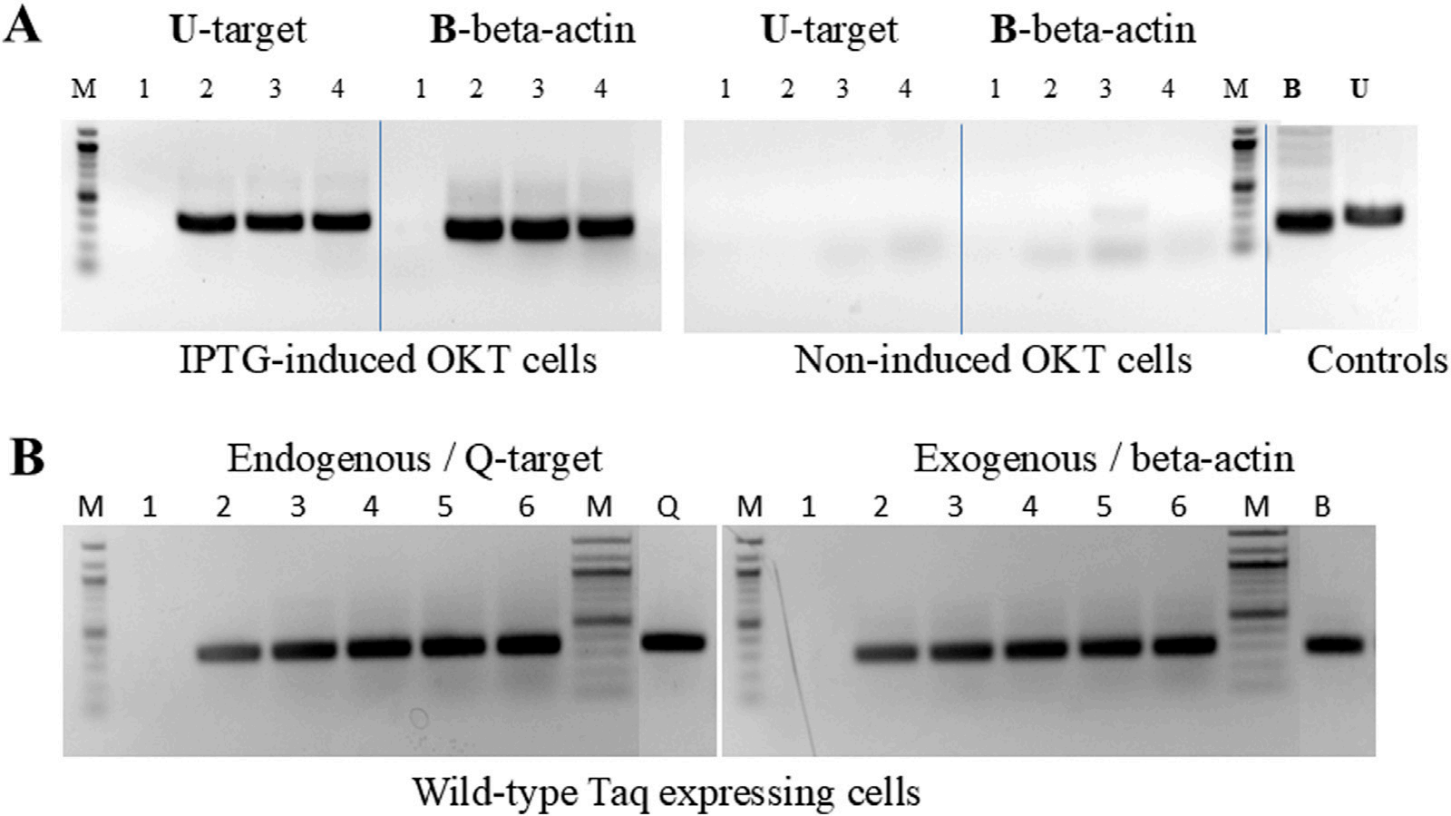
“Live Culture PCR” amplification of endogenous and exogenous DNA targets. **(A)** The endogenous bacterial rDNA targetU, used in Figure 2, was amplified in the presence of 0, 2, 4, and 8 µL of cell culture (lanes 1–4) expressing OmniKlentaq, either IPTG-induced or non-induced. The same cells and culture volumes were used to amplify in parallel a 296 bp human beta-actin gene target B, after adding 10 ng of human genomic DNA and beta-actin primers to the reaction. In the positive controls (lanes B and U), the same targets were amplified by conventional PCR with 0.05 µL of purified OKT enzyme, using 10 ng of human DNA and 1 ng of bacterial DNA, respectively. **(B)** The endogenous bacterial rDNA target Q, used in Figure 2, was amplified in the presence of 0, 2, 4, 6, 8, and 10 µL of cells expressing wild-type (w.t.) Taq (lanes 1–6). The same cells and culture volumes were used to catalyze the human beta-actin gene target B, used in Figure 3, from 10 ng of human genomic DNA. In the positive controls (lanes Q and B), the same targets were amplified by conventional PCR with 0.5 µL of purified Taq enzyme, using 200 pg of bacterial DNA and 10 ng of human DNA, respectively. Amplified products were analyzed by 1.5% agarose gel electrophoresis, stained with ethidium bromide. Lanes M, 100 bp DNA ladder.

We also tested the option to amplify an exogenous, non-bacterial target directly with cells providing the enzyme. A human beta-actin gene target was efficiently amplified, with amplification levels identical to conventional PCR using purified enzyme and DNA template. This suggests that the application scope of LC-PCR can be expanded (Figure 3A).

In another set of experiments, we successfully applied the LC-PCR protocol to wild-type Taq-expressing cells (Figure 3B). These cells could efficiently amplify both endogenous and exogenous targets, indicating that the procedure is not limited to inhibition-resistant Taq mutants like OT and OKT. The amplification profiles were comparable to control reactions performed with purified enzymes and DNA templates. This sets the stage for developing an LC-PCR protocol for functional screening of libraries of randomly mutagenized Taq enzymes.

### Direct cell culture real-time PCR screening of mutagenized Taq and Klentaq1 libraries

**FIGURE 4:**
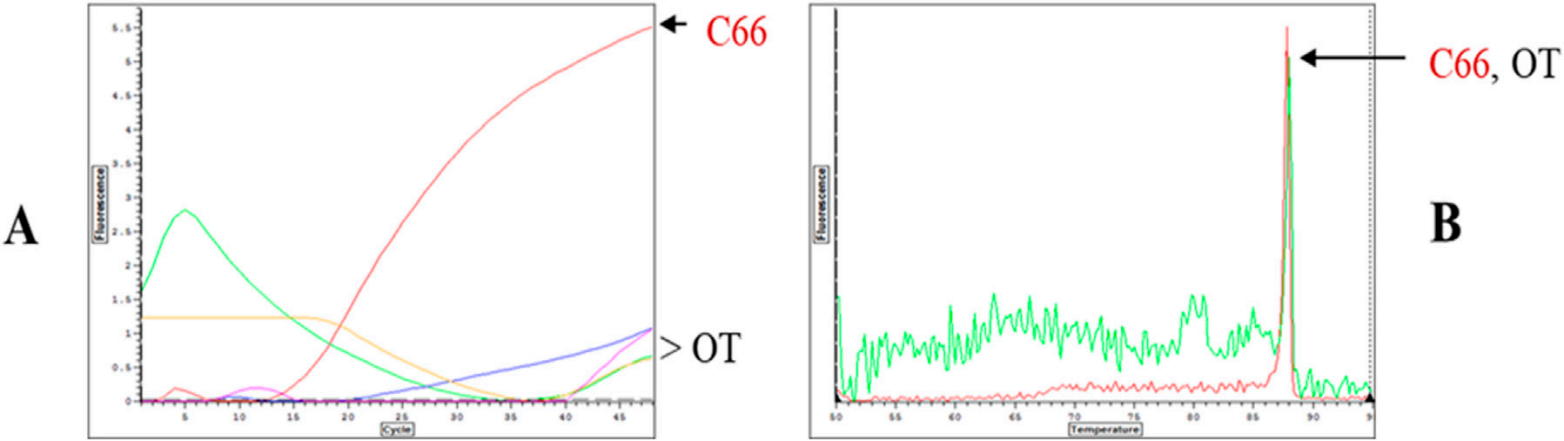
“Live Culture PCR” screening of a library of mutagenized Taq pol by direct real-time amplification with whole bacterial cells harboring individual mutant clones. Single colonies from a randomly mutagenized Taq pol library were grown and IPTG-induced overnight in individual wells of 96-well plates. Five µL of cells from each well were then transferred to a replica (PCR) 96-well plate containing 30 µL of PCR buffer, dNTPs, primers for rDNA U target, 0.5X SYBR Green, and the challenging PCR inhibitor (2 µL of 10% chocolate per reaction). The replica plates were directly subjected to real-time PCR. The corner wells contained OmniTaq (OT)-expressing cells as controls. No enzyme or DNA were added, as both the enzyme and template were provided by the library cells. **(A,B)** Amplification and melting curves of clone C66 and OmniTaq alone, extracted from the plate data. The pink curve corresponds to an inhibition-resistant clone C66.

Building on the initial data with LC-PCR, we developed and validated a rapid real-time PCR library screening protocol. In this protocol, intact library cells harboring individual clones of wild-type Taq pol or Klentaq1 were used to catalyze amplification of their own rRNA genes in a 96-well plate format. By incorporating PCR inhibitors in the master mix, we were able to select inhibition-resistant mutants of Taq and Klentaq1. The screening protocol’s value was demonstrated by selecting a novel Taq mutant, C-66, based on its high tolerance to chocolate, a potent PCR inhibitor (Kacli et al., 2007). The best-performing inhibition-resistant mutants were identified by their lowest Ct values (threshold cycle), indicative of the least inhibition (Figure 4).

A separate screening of the same 96-well plate showed that C-66 also exhibited resistance to black pepper, another highly inhibitory substance in food safety tests (Dhanya et al., 2007). Additionally, using the same procedure on a mutagenized Klentaq1 library, we selected a Klentaq1 mutant, H101, which was highly resistant to these two food inhibitors. Sequence analysis revealed different amino acid changes: E818V in C-66, and K738R in H101(Supplementary Data).

### Selected Taq and Klentaq1 inhibition-resistant mutants, C-66 and H101, allow efficient PCR in the presence of various PCR inhibitors

**FIGURE 5:**
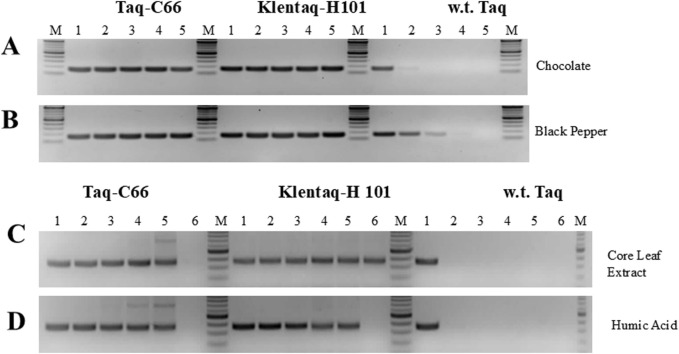
PCR in the presence of various inhibitors with the novel Taq pol mutants C66 and H101. **(A,B)** A 160 bp *Salmonella* HIL-A gene target was amplified with Taq-C66, Klentaq1-H101, or wild-type Taq from 1 ng *Salmonella* DNA for 30 cycles in the presence of 0, 0.015, 0.03, 0.06, or 0.125 µL of a 10% dark chocolate (70% cocoa) solution, or a 25% black pepper solution (lanes 1–5, respectively). **(C,D)** A 360 bp soybean cyst nematode (SCN) target was amplified from 5 ng SCN DNA with Taq-C66, Klentaq1-H101, or wild-type Taq for 40 cycles in the presence of 0, 0.25, 0.5, 1.0, 2.0, or 4.0 µL of crude corn leaf extract, or 0, 31, 63, 125, 250, or 500 ng of humic (lanes 1–6, respectively). The enzyme amounts used in 50 µL reactions with all inhibitors were 0.2 µL Taq-C66, 0.2 µL Klentaq1-H101, and 0.4 µL wild-type Taq. No PCR enhancer was used. Amplified products were analyzed by 2.5% agarose gel electrophoresis, stained with ethidium bromide. Lanes M, DNA ladder standards.

**FIGURE 6:**
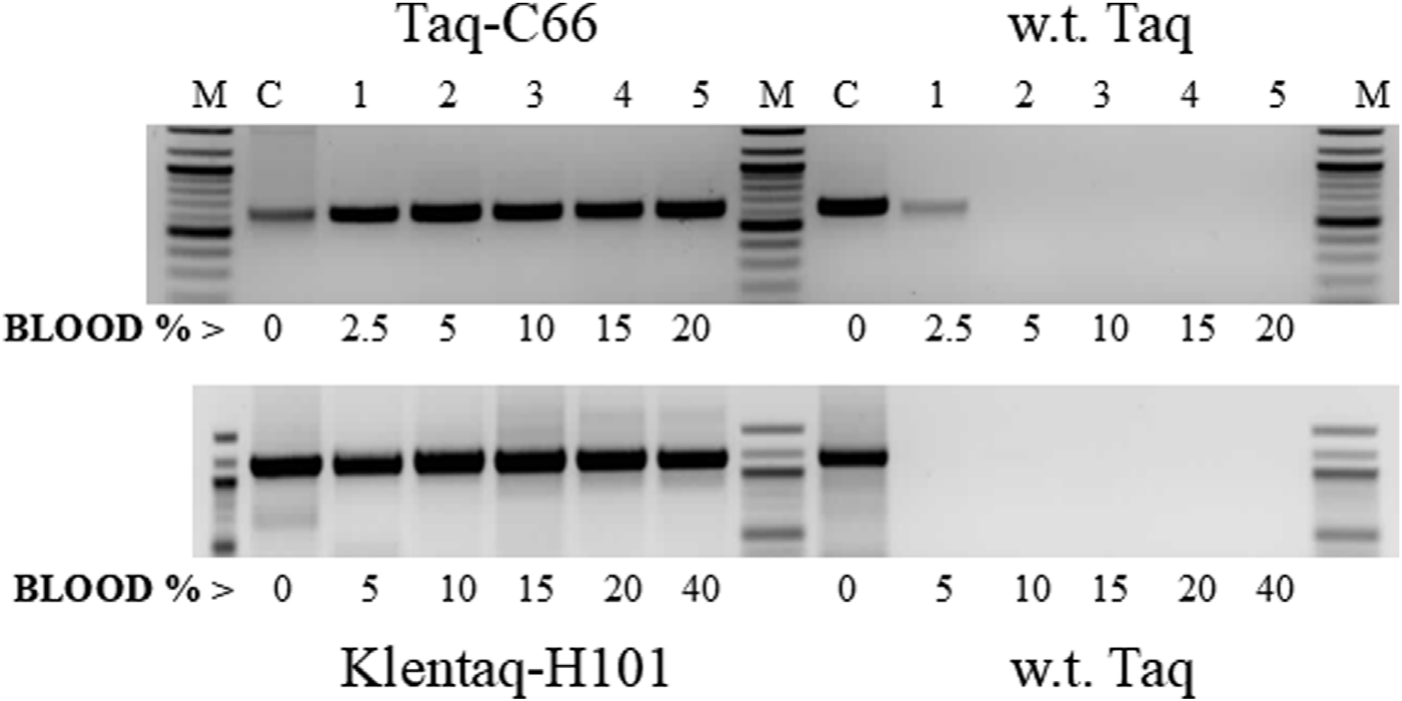
Direct PCR in high blood concentrations with the mutant enzymes Taq-C66 and Klentaq1-H101. Two targets of the human HIV-receptor gene CCR5, 630 bp (top) and 1.1 kb (bottom), were amplified for 35 cycles directly from 2.5% to 40% human blood (lanes 1–5) with the mutant enzymes Taq-C66 or Klentaq1-H101, as well as wild-type Taq. Control reactions (lanes C) contained no blood, but 10 ng of human DNA. The enzyme amounts used in 50 µL reactions were 0.3 µL Taq-C66 vs. 0.6 µL wild-type Taq (top) and 0.5 µL Klentaq1-H101 vs. 1.25 µL wild-type Taq (bottom). Reactions contained no PCR enhancer. Amplified products were analyzed by 1.5% agarose gel electrophoresis, stained with ethidium bromide. Lanes M, DNA ladder standards.

The inhibitor tolerance phenotype was confirmed with both mutant enzymes after they were purified from the selected clones. These mutants displayed high tolerance to both chocolate and black pepper at concentrations that significantly inhibited or completely inhibited wild-type Taq (Figure 5). Moreover, these mutants showed strong resistance to a variety of PCR inhibitors found in blood, humic acid, plant tissues, and bile salts, demonstrating their suitability for direct PCR from crude clinical, environmental, and forensic samples (Figures 5, 6).

## Discussion

In this study, we aimed to develop a simple and time-saving protocol for the functional screening of randomly mutagenized libraries of Taq DNA polymerase. We discovered that bacterial cells overexpressing wild-type Taq pol or its mutant forms can be used directly in PCR without enzyme purification, skipping cell washing or lysis steps. We develop a method to screen a library to identify inhibition-resistant Taq and Klentaq mutants. The cells, providing both enzyme and template, can efficiently amplify both endogenous and exogenous DNA targets.

We applied this “live culture” PCR (LC-PCR) method for screening Taq pol and Klentaq1 mutant libraries. The protocol was optimized for real-time PCR in a microtiter plate format, where PCR inhibitors were incorporated into the reaction to positively select inhibition-resistant polymerases. Fortunately, we discovered two standout mutants, H101 and C66 (now commercialized as OmniTaq 3 and Omni Klentaq 2, respectively), after screening only ∼4,000 and ∼10,000 colonies, respectively. Although continued screening yielded additional promising mutants, none matched the inhibitor resistance of H101 and C66. The selected mutants, C-66 and H101, exhibited remarkable resistance to PCR inhibitors, making them suitable for direct PCR assays on crude clinical, environmental, or food safety samples without DNA extraction.

Our findings indicate that the LC-PCR procedure can be adapted to screen for other desirable polymerase traits, such as elongation speed, by tuning the selective PCR parameters. Using this approach, we recently identified a high-speed Taq mutant capable of amplifying DNA at 1 kb per second (manuscript in preparation). We also discovered several bifunctional Taq mutants capable of amplifying both DNA and RNA targets.

One intriguing finding with the mutants C66 and H101, described here, was their concomitant resistance to a variety of PCR inhibitory samples. It is plausible that a spectrum of phenolic and polyphenolic compounds could be among the common inhibitory substances found in chocolate, black pepper, humic acid, and plant extracts (Kacli et al., 2007; Dhanya et al., 2007; Watson and Blackwell, 2000; Rezadoost et al., 2016; Schrader et al., 2012). The co-resistance of these mutants to other potent PCR inhibitors, such as blood components (hemoglobin, lactoferrin, IgG) and bile salts (Akane et al., 1994; Al-Soud et al., 2000; Al-Soud and Rådström, 2001), suggests that all the tested inhibitory substances may act through a similar mechanism—potentially targeting a common site in the Taq polymerase enzyme, DNA, or both.

**FIGURE 7:**
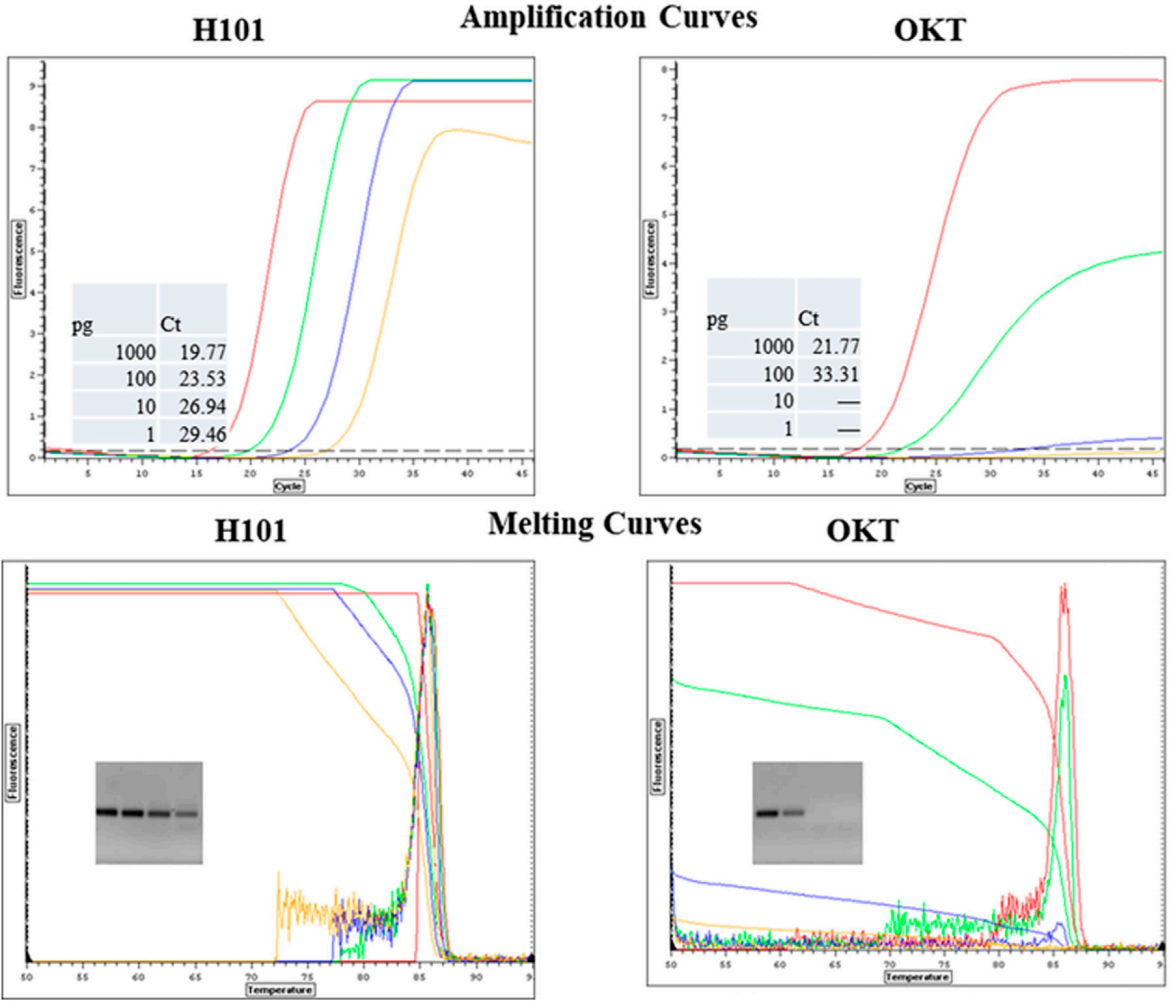
Comparison of Mutant H101 and OKT for Resistance to Chocolate in qPCR. *Salmonella* DNA was 10-fold serially diluted from 1000 pg to 1pg and it was detected by qPCR with SYBR Green with primers for a HiLA-3 gene. The reactions included 0.6 µL of purified Omni Klentaq (OKT) or the inhibition-resistant mutant H101 with 2 µL 10% chocolate extract per 35 µL reaction.

Since the inhibitory samples represent complex mixtures, the exact mechanism(s) of inhibition and tolerance remain to be elucidated in future studies with purified inhibitors. Previously, we reported that mutations in Taq codon 708 (mutants E708K and E708Q, now commercially available as “OmniKlentaq” and “OmniTaq”) confer resistance to a similar panel of inhibitors (Lawyer et al., 1989). In comparative assays, the novel Taq polymerase and Klentaq1 mutants described here proved superior to the 708 mutants in all aspects of inhibition resistance (example is shown in Figure 7).

**FIGURE 8:**
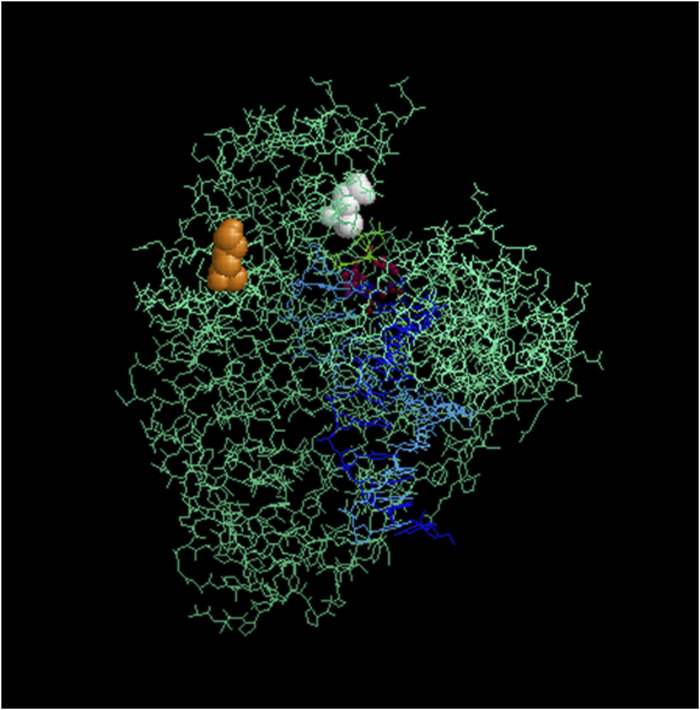
Location of the mutagenized codons in Taq/Klentaq1. Crystal structure of Klentaq1 from PDB file 2KTQ emphasizing the location of the residues mutated in Klentaq1 H101 and Taq-66. These residues are rendered in space-fill for the mutated positions 738 (orange) and 818 (white), using the program Rasmol 2.7.5 (Sayle and Milner-White, 1995). Residues 610, 785, and 786, which are involved in the active site, are shown in burgundy. The primer and template DNA strands are depicted in blue. Upper right: thumb domain; upper left: fingers domain.

Codon K738 lies at the edge of the alpha-helix Q region of the “fingers domain” in Taq and plays a role in nucleotide interaction within the closed ternary enzyme complex (Li et al., 1998; Doublié et al., 1999) (Figure 8). In the Klentaq1 H101 mutant (K738R), the increased positive charge at this site may enhance the enzyme’s affinity for incoming nucleotides and/or stabilize the template–enzyme complex. This region is surrounded by “finger” residues interacting with DNA, such as 728Y, 746R, 747M, and 750N (Al-Soud and Rådström, 2001; Li et al., 1998). Notably, adjacent mutations near this site, E742R and A743R, also showed increased DNA-binding affinity and enhanced primer extension activity (Yamagami et al., 2014). These findings raise the possibility that specific interactions within this region could be disrupted by inhibitors that compete with nucleotide or DNA binding.

Codon E818, implicated in the phenotype of the Taq-66 mutant (E818V), is located within the “palm” subdomain near the C-terminus (Sayle and Milner-White, 1995). (Sayle and Milner-White, 1995) (Figure 8). Although the structural impact of this mutation remains speculative, the notable resistance of this variant to PCR inhibitors suggests that a change in local polarity may induce a conformational shift that reduces inhibitor sensitivity. To explore these possibilities, we plan to conduct steady-state kinetic assays to assess changes in catalytic efficiency and DNA-binding assays to quantitatively evaluate differences in template affinity. These biochemical studies will provide mechanistic insight into how these mutations contribute to improved inhibitor resistance.

## Data Availability

The amino acid sequences generated in this study are provided in the Supplementary Material. The other datasets generated and analyzed for this study are available in the Zenodo repository at: https://doi.org/10.5281/zenodo.16852658.
