# Supplementary material for: Live culture-based qPCR screening of Taq DNA polymerase variants for resistance to PCR inhibitors
Source: Front Bioeng Biotechnol. 2025 Aug 29;13:1624735. doi: 10.3389/fbioe.2025.1624735 (PMC12425957; doi:10.3389/fbioe.2025.1624735)
Supplement: Supplementary file 1 [file DataSheet1.pdf]

MRGMLPLFEPKGRVLLVDGHHLAYRTFHALKGLTTSRGEPVQAVYGFAKSLLKALKEDGDAVIVVFDAKAPSFRHEAYGG

YKAGRAPTPEDFPRQLALIKELVDLLGLARLEVPGYEADDVLASLAKKAEKEGYEVRILTADKDLYQLLSDRIHVLHPEGYLIT

PAWLWEKYGLRPDQWADYRALTGDESDNLPGVKGIGEKTARKLLEEWGSLEALLKNLDRLKPAIREKILAHMDDLKLSW

DLAKVRTDLPLEVDFAKRREPDRERLRAFLERLEFGSLLHEFGLLESPKALEEAPWPPPEGAFVGFVLSRKEPMWADLLAL

AAARGGRVHRAPEPYKALRDLKEARGLLAKDLSVLALREGLGLPPGDDPMLLAYLLDPSNTTPEGVARRYGGEWTEEAG

ERAALSERLFANLWGRLEGEERLLWLYREVERPLSAVLAHMEATGVRLDVAYLRALSLEVAEEIARLEAEVFRLAGHPFNL

NSRDQLERVLFDELGLPAIGKTEKTGKRSTSAAVLEALREAHPIVEKILQYRELTKLKSTYIDPLPDLIHPRTGRLHTRFNQTA

TATGRLSSSDPNLQNIPVRTPLGQRIRRAFIAEEGWLLVALDYSQIELRVLAHLSGDENLIRVFQEGRDIHTETASWMFGV

PREAVDPLMRRAAKTINFGVLYGMSAHRLSQELAIPYEEAQAFIERYFQSFPKVRAWIEKTLEEGRRRGYVETLFGRRRYV

PDLEARVKSVREAAERMAFNMPVQGTAADLMKLAMVKLFPRLEEMGARMLLQVHDELVLEAPKERAEAVARLAKEV

MEGVYPLAVPLVVEVGIGEDWLSAKE

**Amino acid sequence of the C66 Taq polymerase mutant.** The full-length amino acid sequence of the Taq DNA polymerase C66 mutant is shown. The gene encodes an 832-amino-acid protein. The C66 mutant carries a single amino acid substitution (E818V), which is highlighted in the sequence.

--------------------------------------------------------------------------------------------------------------------------------------------

MGLLHEFGLLESPKALEEAPWPPPEGAFVGFVLSRKEPMWADLLALAAARGGRVHRAPEPYKALRDLKEARGLLAKDLS

VLALREGLGLPPGDDPMLLAYLLDPSNTTPEGVARRYGGEWTEEAGERAALSERLFANLWGRLEGEERLLWLYREVERPL

SAVLAHMEATGVRLDVAYLRALSLEVAEEIARLEAEVFRLAGHPFNLNSRDQLERVLFDELGLPAIGKTEKTGKRSTSAAVL

EALREAHPIVEKILQYRELTKLKSTYIDPLPDLIHPRTGRLHTRFNQTATATGRLSSSDPNLQNIPVRTPLGQRIRRAFIAEEG

WLLVALDYSQIELRVLAHLSGDENLIRVFQEGRDIHTETASWMFGVPREAVDPLMRRAAKTINFGVLYGMSAHRLSQEL

AIPYEEAQAFIERYFQSFPKVRAWIEKTLEEGRRRGYVETLFGRRRYVPDLEARVRSVREAAERMAFNMPVQGTAADLM

KLAMVKLFPRLEEMGARMLLQVHDELVLEAPKERAEAVARLAKEVMEGVYPLAVPLEVEVGIGEDWLSAKE

**Amino Acid Sequence of the H101 Taq Polymerase Mutant**. The H101 mutant is a truncated variant of Taq DNA polymerase, lacking the N-terminal 278 amino acids present in the full-length enzyme. The sequence shown corresponds to the complete amino acid sequence of the H101 mutant, a 554-residue protein derived from the Klentaq1 version of Taq polymerase.

The H101 mutant contains a single point mutation, K738R (lysine to arginine), where the residue position is numbered according to the full-length Taq DNA polymerase. The mutated residue is highlighted in the sequence.
